# A Rare Cause of Dyspnea in a Patient Presenting to the Emergency Department: A Case Report and Review of the Literature of Laparoscopic Cholecystectomy Complication

**DOI:** 10.4021/jocmr664w

**Published:** 2011-09-26

**Authors:** Mehmet Okumus, Fikret Ezberci, Nuretdin Kuzhan, Eyup Mehmet Pircanoglu, Selim Bozkurt

**Affiliations:** a Faculty of Medicine, Kahramanmaras Sutcuimam University, Emergency Department, Kahramanmaras, Turkey; b Faculty of Medicine, Kahramanmara? Sutcuimam University, General Surgery Department, Kahramanmaras, Turkey; c Faculty of Medicine, Kahramanmaras Sutcuimam University, Infection Disease and Clinic Microbiology Department. Kahramanmaras, Turkey

## Abstract

In recent years, there has been an emerging preference by patients for emergency services due to the efficiency of transactions and the increased quality of service. As a result, emergency departments have experienced an extreme density of patients. Nevertheless, patients require the same standard of care and should be evaluated carefully. The emergency physician should evaluate the patient as a whole in light of the findings and not merely according to the recommendations of the consultant. We describe in this case report a rare complication occurring after laparoscopic cholecystectomy, shortness of breath, which could easily be disregarded.

**Keywords:**

Laparoscopic cholecystectomy complication; Dyspnea; Intrahepatic subcapsular hematoma

## Introduction

The open cholecystectomy surgical procedure was widely used previously. However, since its development in 1987, laparoscopic cholecystectomy (LC) is becoming more common [1]. Due to its minimal invasiveness, limited incision sites and the short hospitalization period, LC has become a preferred application by both surgeons and patients [2]. While LC is a very safe procedure in selected cases [3], complications such as bile leakage, wound infection, bleeding, bile duct stones, cardiovascular complications, respiratory complications, bowel injury, postoperative acute pancreatitis, omental hematoma, hematoma of the abdominal wall, port site herniation, common bile duct incision, subhepatic abscess, and subcapsular hematoma can be seen rarely. Subcapsular hematoma is a rare and uncommon complication of LC [4]. The frequency of intrahepatic subcapsular hematoma (ISH) without bleeding into the peritoneum is about 0.08% to 0.2% [5]. In this article, we will discuss the incidence, differential diagnosis and treatment of this late complication after LC.

## Case Report

**Figure 1:**
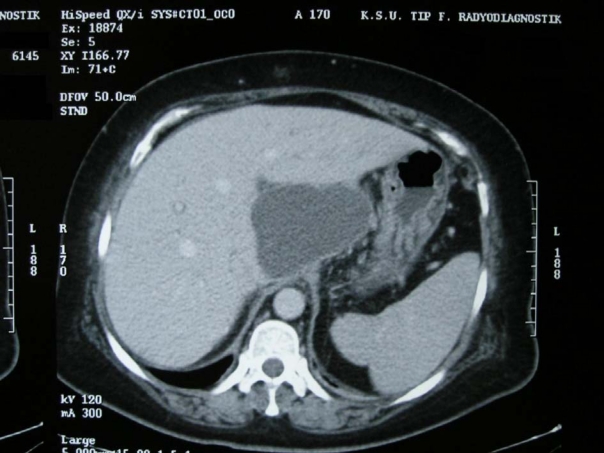
Intrahepatic hematoma at size 19 x 12 x 5 cm has shown by abdominal computerized tomography

A 59-year-old female presented to the emergency department with the complaint of shortness of breath. She had undergone LC 20 days before, following which she began to experience shortness of breath. Due to her normal vital signs, the complaint of the patient was considered to be associated with the operation and she was discharged with an analgesic (20 mg Tenoxicam). Her symptoms had increased over time after discharge. Because of fever and increased dyspnea, the patient presented to the emergency room. Her physical examination revealed: normal TA (110/70 mmHg), fever (38 °C) and tachycardia (100/min). Ecchymosis of approximately 15 x 20 cm was noted in the periumbilical region, and bowel sounds were normal. Her abdomen was soft with tenderness on palpation, with widespread tenderness over the right hypochondriac region. Hepatomegaly was noted, with the liver palpable approximately 8 cm below the costal margin. Laboratory tests demonstrated white cell count of 16.4 x 10^9^/L, and blood count revealed decreased hemoglobin (8.9 mg/L) and increased C-reactive protein (CRP, 144 mg/L). The liver function tests were unremarkable. The patient's respiratory and cardiac systems were examined and no abnormalities were determined. To ascertain the source of anemia and fever, an abdominal computerized tomography (CT) was performed, which demonstrated intrahepatic fluid collection measuring approximately 19 x 12 x 5 cm extending from the portal hilum to the posteroinferior segment (Fig. 1); there was no free fluid in the peritoneal cavity.

**Figure 2:**
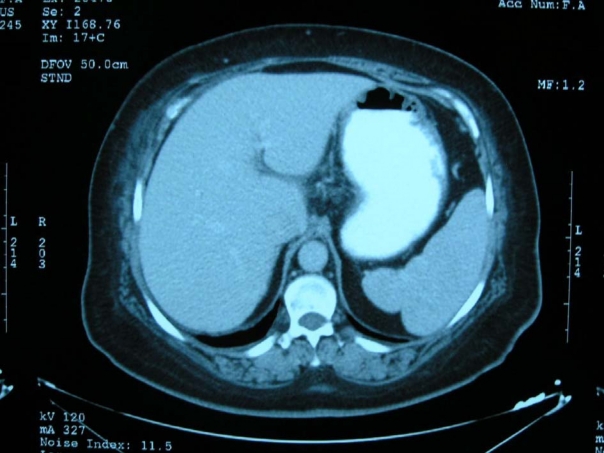
Two months later abdominal computerized tomography has shown resolution of the intrahepatic hematoma

Percutaneous aspiration of the intrahepatic fluid collection under ultrasound guidance was performed. The aspiration revealed hemorrhagic fluid that was not coagulated. *Escherichia coli* was isolated from the aspirate culture, which was taken from fluid collection. The patient was given ceftriaxone 1 g twice daily intravenously and two units of erythrocyte suspension. Ceftriaxone therapy was continued for 14 days. During her hospitalization, there was no change in the hematoma throughout the ultrasonography (USG) follow-up. The patient improved and was discharged with normal laboratory and vital findings. Follow-up abdominal CT examination two months later showed almost complete resolution of the hematoma (Fig. 2).

## Discussion

Readmission to the hospital following LC due to ISH is uncommon in the literature. A literature search revealed only a few cases of ISH without hemoperitoneum that occurred after LC.

Unruptured ISH without peritoneal bleeding is extremely rare, as in this case, and the literature describes only six cases [6-11]. The sources of bleeding during LC are trocar sites, the liver bed, and vascular injury, and the bleeding is usually observed and homeostasis achieved during the operation [12-14]. Although ISH is a rare complication after LC, the situation may be life-threatening if left untreated. The cause of ISH after LC is not clearly known in all cases. Three causes were described in a report on liver hematoma: puncture of the liver with the trocar during its placement into the abdomen, parenchymal injury during excision of the gallbladder and excessive bending and wrinkling of the liver capsule during retraction and dissection of the gallbladder [15]. Aside from surgical applications, some drugs such as non-steroidal anti-inflammatory drugs (NSAIDs) are cited as responsible for ISH. In two cases, ISH occurred after LC when Ketorolac was used for pain control. The exploratory laparoscopy revealed no apparent hepatic injury other than ISH, and Ketorolac was determined to be responsible due to its strong antiplatelet activity [16,17]. The patients with ISH after LC mostly complain of pain or discomfort in the right upper quadrant, and physical examination reveals hypotension and tachycardia. The laboratory studies show a decrease in hemoglobin levels in most patients [6-17]. However, in this case, the vital signs were within normal ranges and the main complaint was shortness of breath; the hemoglobin level was not severely decreased, as seen in some other cases. If the subcapsular hematoma is not accompanied with rupture, the hematoma is small, and the patient is stable and asymptomatic, a conservative therapy may be possible. If the hematoma appears to be of a size that can be reabsorbed spontaneously and there is no hemoperitoneum, close observation of an ISH is safe [18]. In the case of persistent abdominal pain, the ISH can be treated by CT/USG-guided drainage, but if the patient’s condition is severe and shock presents accompanied with rupture, surgical intervention may be necessary [17, 19-21]. In the present case, close observation was preferred with follow-up USG examination in view of the patient’s stable condition and because her vital signs were normal and she responded to the treatment and improved.

In conclusion, this case report describes a very rare complication and different complaint following LC, shortness of breath. LC remains a very safe application in selected patients. However, ISH may occur in patients with right upper quadrant discomfort, hypotension and tachycardia. In some patients with persistent abdominal discomfort or unexpected postoperative signs or symptoms after LC, the physician should be aware of the possibility of ISH.
